# Radiomic Profiling of Tumor Thrombus for Predicting Recurrence in Renal Cell Carcinoma

**DOI:** 10.1016/j.euros.2025.06.005

**Published:** 2025-07-25

**Authors:** Zine-Eddine Khene, Isamu Tachibana, Raj Bhanvadia, Ivan Trevino, Prajwal Sharma, William Graber, Nicholas Bingham, Theophile Bertail, Raphael Fleury, Kris Gaston, Solomon L. Woldu, Karim Bensalah, Yair Lotan, Vitaly Margulis

**Affiliations:** a Department of Urology, UT Southwestern Medical Center, Dallas, TX, USA; b Department of Urology, University of Rennes, Rennes, France; c Image and Signal Processing Laboratory, Inserm U1099, University of Rennes, Rennes, France

**Keywords:** Kidney cancer, Renal cell carcinoma, Radiomics, Tumor thrombus, Machine learning, Survival

## Abstract

**Background and objective:**

Clear cell renal cell carcinoma (ccRCC) with tumor thrombus (TT) presents a significant prognostic challenge due to its high recurrence risk. Radiomics, an imaging-based biomarker approach, has primarily focused on the primary tumor, while the prognostic potential of TT radiomics remains largely unexplored. This study aimed to assess the added value of tumor thrombus radiomic signatures (RSs) in predicting recurrence in ccRCC patients with TT.

**Methods:**

We conducted a retrospective analysis of patients undergoing surgical resection for nonmetastatic ccRCC with TT. Preoperative contrast-enhanced computed tomography images were used to extract radiomic features from the primary tumor and TT. Features were selected using least absolute shrinkage and selection operator (LASSO) Cox regression and incorporated into predictive models. Performance was assessed using the integrated area under the curve (iAUC), calibration, decision curve analysis (DCA), and incremental value over clinical models (pTNM, UISS, and Leibovich) for the prediction of disease-free survival (DFS).

**Key findings and limitations:**

A total of 166 patients (training set: *n* = 117; test set: *n* = 49) were included. The primary tumor RS achieved an iAUC of 0.69, the TT RS achieved 0.78, and the primary tumor + TT RS achieved 0.82. Incorporation of TT radiomics enhanced the predictive accuracy of clinical models significantly, with iAUC increases from 0.58 to 0.83 for pTNM, 0.64 to 0.83 for UISS, and 0.66 to 0.83 for Leibovich scores (all *p* < 0.001). The DCA confirmed the clinical utility of integrating radiomic features, particularly TT radiomics, into recurrence risk assessment. The retrospective design and absence of external validation in independent, multicenter cohorts limit the generalizability of these findings.

**Conclusions and clinical implications:**

Tumor thrombus radiomic profiling improves DFS prediction significantly and adds complementary prognostic value to established models in patients with ccRCC. Incorporation of these features into clinical workflows may enhance risk stratification and guide personalized treatment planning. Prospective validation in large, multicenter cohorts is warranted to support clinical adoption.

**Patient summary:**

This study focused on kidney cancer with tumor thrombus and demonstrated that an analysis of the imaging features from the thrombus improved the prediction of cancer recurrence significantly. This approach could enhance the understanding of individual patient risks and support more personalized treatment strategies.

## Take Home Message

Integration of radiomic features from the tumor thrombus improves recurrence prediction in patients with clear cell renal cell carcinoma and tumor thrombus. This approach provides valuable insights into individual patient risk profiles and can facilitate more personalized treatment strategies.

## 1 Introduction

Renal cell carcinoma (RCC) with venous involvement (extension into the renal vein or inferior vena cava) is observed in 4–10% of newly diagnosed RCC cases [1]. For patients with nonmetastatic disease, surgical removal of the primary tumor and thrombus is the standard curative treatment [2]. However, this procedure is associated with significant perioperative morbidity and a high recurrence rate, with the overall survival rate ranging from 40% to 65% at 5 yr for patients with tumor thrombus (TT) [3].

Although the TNM staging system and pathological grade are used commonly for recurrence risk stratification, considerable variability in patient outcomes persists even among individuals with similar stages and grades [4]. This discrepancy highlights the limitations of current staging systems in accurately predicting oncological outcomes. Addressing these limitations requires more precise risk stratification to identify patients who may benefit from intensified treatment, closer monitoring, or adjuvant therapy [5].

Quantitative imaging techniques known as radiomics have emerged as promising tools for identifying imaging biomarkers [6]. By extracting features from regions of interest on imaging studies, radiomics has the potential to refine prognostic accuracy and guide personalized treatment decisions [7]. Although existing radiomic models based on primary renal tumors have demonstrated prognostic value in RCC [8], the potential of radiomics from TT remains unexplored, despite biological evidence of distinct phenotypes between the primary tumor and associated thrombus [9].

We hypothesized that incorporation of radiomic features from TT could enhance recurrence prediction and improve individualized patient stratification. Therefore, this study aimed to evaluate the incremental prognostic value of a radiomic signature (RS) derived from TT imaging in patients with clear cell RCC (ccRCC).

## 2 Patients and methods

### 2.1 Study design and participants

Following institutional review board approval (STU-2023-1172), we conducted a retrospective analysis of all patients who underwent surgical resection of nonmetastatic ccRCC with venous TT (renal vein, inferior vena cava, and right atrium) and had available preoperative diagnostic computed tomography (CT) scans, between January 1, 2015, and December 31, 2022, at two medical centers. Patients were included if they met the following criteria: (1) histologically confirmed nonmetastatic ccRCC, (2) venous TT, and (3) postoperative follow-up data. A postoperative histopathological evaluation was performed for all patients to confirm the diagnosis of ccRCC and TT. Patients were excluded if they received adjuvant or neoadjuvant therapy (*n* = 35) or lacked postoperative follow-up data (*n* = 6). Initially, 207 patients met the inclusion criteria; after applying the exclusion criteria, 166 patients remained for the analysis.

### 2.2 Follow-up protocol and outcomes

Postoperative follow-up was institution and physician dependent, but generally followed national and international guidelines. It usually comprised an outpatient visit at 1 mo postoperatively, then every 6 mo for 3 yr and annually for at least 3 additional years. Follow-up consisted of a physical examination and contrast-enhanced CT of the chest, abdomen, and pelvis. Recurrence was determined based on the identification of new local or distant lesions on CT imaging, as interpreted by board-certified radiologists, and confirmed by biopsy when clinically feasible. The endpoints of interest were disease-free survival (DFS). DFS was defined as the time from surgery to disease recurrence (including local and distant recurrences) or death from any cause. Patients alive without recurrence were censored at the date of their last follow-up.

### 2.3 Imaging acquisition and tumor segmentation

Abdominal contrast-enhanced CT was performed presurgically; details of the CT scanners and parameters are provided in Supplementary Table 1. Images were normalized using the 3σ method and reconstructed to a 5-mm slice thickness. To address variability in CT vendors and acquisition protocols, Bootstrapped ComBat (B-ComBat) harmonization was applied [10]. TT and primary renal masses were segmented using a semiautomated method by two physicians using the Fast GrowCut Effect extension in 3D Slicer version 5.6.1 [11]. The representative volumes for the primary tumor and TT are shown in Supplementary Fig. 1.

### 2.4 Radiomic procedure

**Fig. 1:**
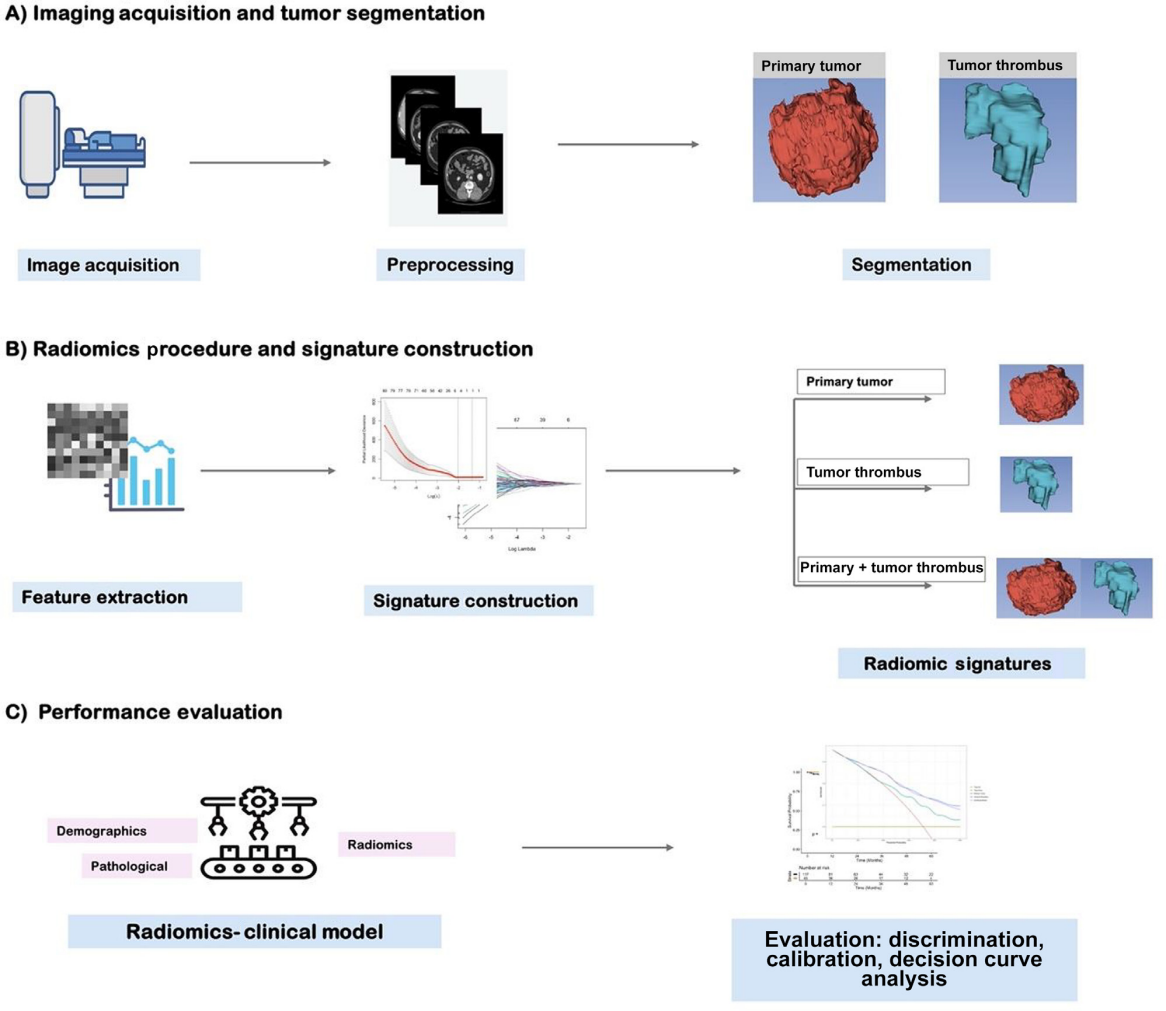
Overall workflow and pipeline of the project.

Fig. 1 shows the radiomic experimental design. Radiomic features were computed using Pyradiomics version 3.0.1 (Imaging Biomarker Standardization Initiative compliant algorithm; Python Software Corporation, Wilmington, DE, USA) [12]. A bin width of 25 was employed. Seven categories of radiomic features were extracted: shape, first order, gray-level co-occurrence matrix, gray-level size zone matrix, gray-level run length matrix, gray-level dependence matrix, and neighboring gray tone difference matrix features. Additionally, Laplacian of Gaussian features (σ = 1–5) and wavelet features with eight decompositions were extracted. In total, 1316 radiomic features were extracted per patient. Detailed information on the radiomic procedure is provided in Supplementary Table 2.

### 2.5 RS construction

Features were extracted from the primary tumor and TT volumes of interest. Intraclass correlation coefficients were calculated, and only parameters with an intraclass correlation coefficient of above 0.75 were included in the analysis [13]. Feature selection was performed using the least absolute shrinkage and selection operator (LASSO) Cox regression algorithm, which identifies the most predictive features by applying regularization to reduce overfitting and eliminate noninformative variables. Features with nonzero coefficients were selected to construct the primary tumor, TT, and primary tumor RS + TT RS. The radiomic score was calculated for each patient as a linear combination of selected features that were weighted by their respective LASSO coefficients: RS = ∑(βi × Xi), where βi represents the LASSO-derived coefficient of the feature and Xi represents the value of the radiomic feature for a given patient.

### 2.6 Statistical analysis

The study population was split randomly into a training cohort (70% of patients) and a test set (30% of patients). RSs were derived from the training set and subsequently applied in the test set. Population characteristics were reported as frequencies and proportions for categorical variables, and as medians with interquartile ranges (IQRs) for continuous variables. Continuous variables were compared using the Mann-Whitney *U* test, and categorical variables were compared using the chi-square and Fisher’s exact tests. DFS was evaluated using the Kaplan-Meier method and compared across groups with the log-rank test. To address the potential for overfitting in the training set, model performance was assessed using bias-corrected integrated area under the curve (iAUC), with 95% confidence intervals (CIs) calculated via bootstrap resampling (1000 bootstrap samples). The iAUC values were derived by integrating the time-dependent AUC values over the specified time range (0.5–36 mo), weighted by survival probabilities at each time point. The iAUC values range from 0 to 1, with 0.5 indicating random prediction and 1 indicating perfect discrimination ability. Calibration of the models was evaluated using calibration plots, which compared the predicted probabilities of recurrence with the observed probabilities at 36 mo. The clinical utility of the model was assessed using a decision curve analysis (DCA) in the test set. To assess the robustness of the primary tumor + TT RS, a sensitivity analysis was conducted in the training cohort, stratified by thrombus location (renal vein vs inferior vena cava). All analyses were conducted using Python version 3.7.2 (Python Software Corporation) and R version 4.1.3 (R Foundation for Statistical Computing, Vienna, Austria; https://www.r-project.org).

## 3 Results

### 3.1 Clinicopathological characteristics

The study included 166 patients, divided into a training (*n* = 117) and a test (*n* = 49) set (Table 1). The median age was 66 yr (IQR: 60–72) in the training set and 67 yr (IQR: 56–73) in the test set. The median tumor size was 7.6 cm (IQR: 6–10) in the training set and 8 cm (IQR: 5.5–10) in the test set. Eastern Cooperative Oncology Group performance status (≥1) was observed in 26% of patients in the training set and 12% in the test set. The median Charlson comorbidity index value was 3 (IQR: 2–4) in the training set and 3 (IQR: 1–5) in the test set.

**Table 1:** Demographic, baseline, and clinicopathological factors

Characteristic	Training (*N* = 117)	Test (*N* = 49)
Age	66 (60, 72)	67 (56, 73)
Sex (male), *n* (%)	84 (72)	34 (69)
Symptom, *n* (%)		
Incidental	53 (45)	23 (47)
Locally symptomatic	40 (34)	22 (45)
Systemically symptomatic	24 (21)	4 (8.2)
Charlson score	3 (2, 4)	3 (1, 5)
ECOG performance-status score ≥1, *n* (%)	30 (26)	6 (12)
Tumor thrombus, *n* (%)		
Renal	64 (55)	33 (67)
Inferior vena cava	53 (45)	16 (33)
Side (right), *n* (%)	56 (48)	28 (57)
Tumor size (cm)	7.6 (6, 10)	8 (5.50, 10)
pT classification, *n* (%)		
3a	64 (55)	33 (67)
3b	47 (40)	12 (24)
3c	4 (3.4)	2 (4.1)
4	2 (1.7)	2 (4.1)
Regional positive lymph nodes (pN), *n* (%)	8 (6.8)	7 (14)
Nuclear grade, *n* (%)		
2	17 (15)	4 (8.2)
3	51 (44)	24 (49)
4	49 (42)	21 (43)
Sarcomatoid features, *n* (%)	12 (10)	5 (10)
Rhabdoid features, *n* (%)	14 (12)	6 (12)
Coagulative necrosis, *n* (%)	64 (55)	29 (59)
Perinephric fat invasion, *n* (%)	46 (39)	19 (39)
Renal sinus invasion, *n* (%)	88 (75)	33 (67)
Urinary collecting system invasion, *n* (%)	29 (25)	9 (18)
Positive surgical margin, *n* (%)	8 (6.8)	3 (6.1)

ECOG = Eastern Cooperative Oncology Group.

TT was located in the renal vein for 55% of the patients in the training set and 67% in the test set, and was located in the inferior vena cava for 45% and 33% of patients, respectively. Most tumors were of a high nuclear grade (International Society of Urological Pathology grade 3 or 4), accounting for 86% in both sets. Nodal involvement was identified in 6.8% of patients in the training set and 9% in the test set.

A total of 89 patients experienced disease recurrence. The median follow-up for patients alive without recurrence was 38 mo (IQR: 15–61) compared with 22 mo (IQR: 9–35) for those with recurrence. The training and test sets were balanced in terms of follow-up duration and recurrence incidence (Supplementary Fig. 2).

### 3.2 Radiomic feature extraction and construction of RSs

**Fig. 2:**
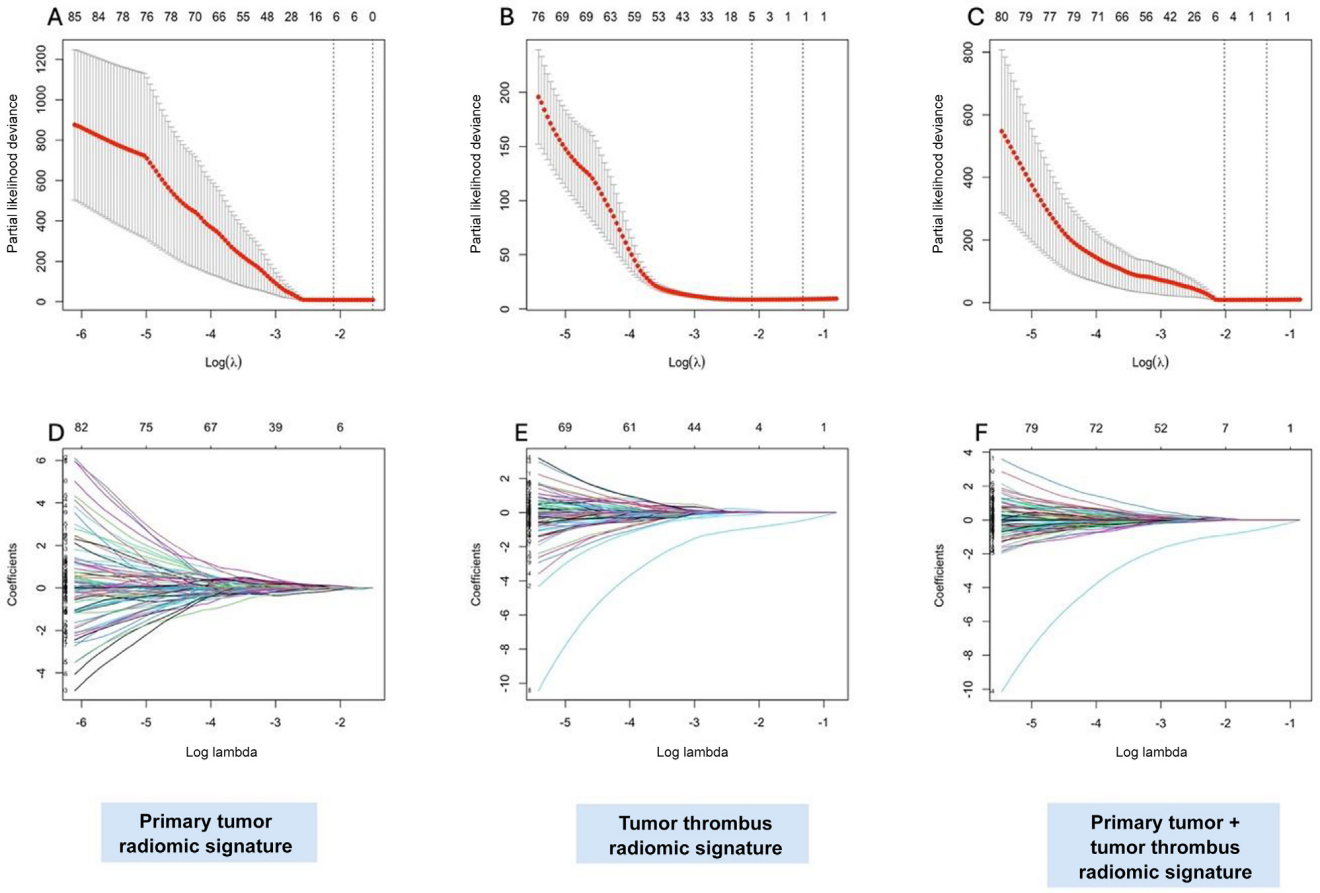
Selection of radiomic features using the LASSO regression model for predicting disease-free survival. Selection of the optimal regularization parameter (λ) in the LASSO Cox regression model for predicting disease-free survival, using ten-fold cross-validation based on minimum criteria. Plots are shown for the (A) primary tumor radiomic signature, (B) tumor thrombus radiomic signature, and (C) combined radiomic signature. LASSO coefficient profiles of the radiomic features for the (D) primary tumor, (E) tumor thrombus, and (F) combined radiomic signatures. The vertical dashed line indicates the selected log(λ) value corresponding to the optimal λ obtained from cross-validation. At this λ, 7, 5, and 7 nonzero coefficients were retained in the primary tumor, tumor thrombus, and combined radiomic signatures, respectively. LASSO = least absolute shrinkage and selection operator.

Initially, 2632 radiomic features were extracted (1316 each from primary tumor and TT volumes). After selecting features based on an intraclass correlation coefficient threshold of >0.75, 728 primary tumor and 785 TT features remained. LASSO Cox regression reduced dimensionality, selecting seven features for the primary tumor model, five features for the TT model, and seven features for the primary tumor RS + TT RS model (Fig. 2). The final RSs and coefficients are provided in Supplementary Tables 3 and 4.

### 3.3 Performance of RSs

The performance of the RSs was evaluated in both the training and the test cohort, as shown in Table 2. In the training set, the primary tumor RS achieved an iAUC of 0.71 (95% CI: 0.61–0.79). In comparison, the TT RS demonstrated a superior iAUC of 0.81 (95% CI: 0.77–0.90). The model incorporating both the primary tumor RS and the TT RS achieved the highest iAUC of 0.86 (95% CI: 0.82–0.91).

**Table 2:** Performance of the radiomic signatures in predicting DFS for RCC with tumor thrombus

	iAUC^a^ (0.5, 36 mo) (95% CI)
*Training cohort (n = 117)*	
Primary tumor RS	0.71 (0.61–0.79)
Tumor thrombus RS	0.81 (0.77–0.90)
Primary tumor RS + tumor thrombus RS	0.86 (0.82–0.91)
*Test cohort (n = 49)*	
Primary tumor RS	0.69 (0.61–0.75)
Tumor thrombus RS	0.78 (0.73–0.83)
Primary tumor RS + tumor thrombus RS	0.82 (0.77–0.86)

AUC = area under the curve; CI = confidence interval; DFS = disease-free survival; iAUC = integrated area under the curve; RCC = renal cell carcinoma; RS = radiomic signature.

a The iAUC values were calculated for the range of 0.5–36 mo using survival-probability weighting and were bias corrected with bootstrap resampling (1000 iterations).

In the test set, similar results were observed: 0.82 (95% CI: 0.77–0.86) for the primary tumor RS + TT RS, 0.78 (95% CI: 0.73–0.83) for the TT RS, and 0.69 (95% CI: 0.61–0.75) for the primary tumor RS. A likelihood ratio test demonstrated that addition of the primary tumor RS to the TT RS improved DFS prediction significantly (*p* = 0.02).

To assess the agreement between predicted and observed recurrence probabilities, calibration was evaluated in the test set using graphical calibration plots at 36 mo (Supplementary Fig. 3). The primary tumor RS model exhibited slight miscalibration in the midrange probabilities, whereas the TT model demonstrated better alignment with the ideal calibration line.

**Fig. 3:**
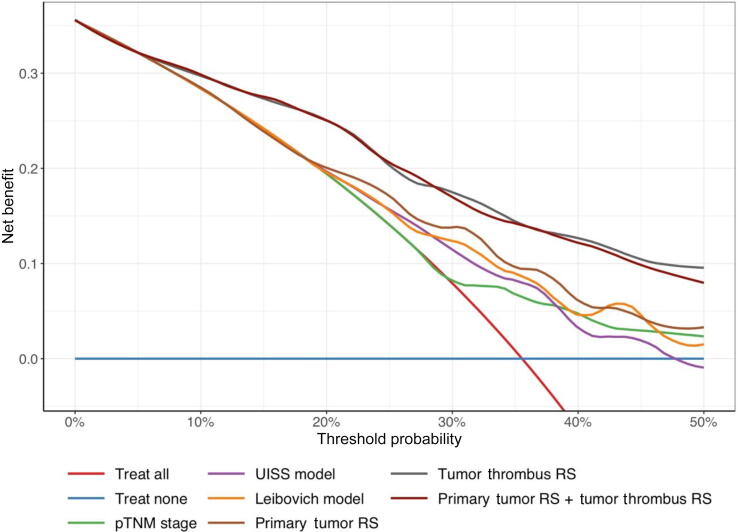
Decision curve analysis of clinical and radiomic models for recurrence prediction in RCC patients with tumor thrombus (test set, 36-mo follow-up). RS = radiomic signature.

Clinical utility of these models was evaluated using a DCA in the test set (Fig. 3). The DCA demonstrated that both the TT RS and the primary tumor RS + TT RS model provided similar net benefit across clinically relevant threshold probabilities (10–50%), outperforming the primary tumor RS and all clinical models.

### 3.4 Incremental value of RSs

We evaluated the added value of RSs by integrating these with clinical models (pTNM stage, UISS, and Leibovich scores) for DFS prediction (Table 3). All combined radiomic clinical models consistently outperformed their respective clinical models alone. Specifically, the iAUC for the TT RS increased from 0.58 to 0.80 when added to the pTNM model, from 0.64 to 0.80 when added to the UISS model, and from 0.66 to 0.80 when added to the Leibovich model. Incorporation of both the primary tumor RS and the TT RS further improved iAUCs to 0.82 with the pTNM, 0.82 with the UISS, and 0.83 with the Leibovich model.

**Table 3:** Integrated AUC (0.5–36 mo) in the test cohort for prognostic scores with and without radiomic signatures^a^, ^b^

Clinical model	Clinical model iAUC (95% CI)	+ Primary tumor RS iAUC (95% CI); *p* value	+ Tumor thrombus RS iAUC (95% CI); *p* value	+ Primary tumor RS and tumor thrombus RS iAUC (95% CI; *p* value)
pTNM stage	0.58 (0.44–0.71)	0.696 (0.603–0.873); 0.001	0.795 (0.701–0.916); <0.001	0.829 (0.785–0.918); <0.001
UISS model	0.64 (0.53–0.74)	0.696 (0.608–0.878); 0.001	0.797 (0.779–0.913); <0.001	0.829 (0.790–0.911); <0.001
Leibovich model	0.66 (0.54–0.75)	0.702 (0.601–0.873); 0.01	0.801 (0.776–0.914); <0.001	0.832 (0.791–0.920); <0.001

AUC = area under the curve; CI = confidence interval; iAUC = integrated area under the curve; RS = radiomic signature.

a For each row, “+ Primary tumor RS/+tumor thrombus RS/+primary tumor RS and tumor thrombus RS” denotes the corresponding clinical baseline combined with the radiomic signature.

b The *p* values are from likelihood ratio tests comparing each “clinical + radiomic” model with its corresponding baseline clinical model (first column).

Finally, a DCA was performed to assess the clinical utility of incorporating radiomics into clinical models. For clarity, we focused on the best performing clinical model (Leibovich score). The DCA showed that the combination of either the TT RS or the primary tumor RS + TT RS with the Leibovich score provided the greatest net benefit across the 10–50% threshold range, outperforming the primary tumor RS + Leibovich model and the clinical model alone (Supplementary Fig. 4).

### 3.5 Sensitivity analysis

To evaluate the robustness of the RSs, a sensitivity analysis was conducted in the training cohort, stratified by thrombus location (renal vein vs inferior vena cava). The predictive performance of both the TT RS and the primary tumor RS + TT RS remained consistent across subgroups and outperformed the primary tumor RS alone (all *p* < 0.05).

## 4 Discussion

In this study, we evaluated three RSs—primary tumor, TT, and primary tumor + TT signatures—derived from preoperative CT scans to predict recurrence in patients with ccRCC and TT. Our results demonstrated that incorporation of TT RSs can enhance the accuracy of recurrence prediction models, suggesting that the thrombus contains biologically distinct information critical to understanding disease progression. While previous studies [[14], [15], [16], [17], [18]] have primarily focused on analyzing the radiomic characteristics of the primary tumor to predict survival outcomes in RCC, our study is the first to investigate the prognostic value of radiomic features specifically extracted from the TT.

The adverse prognosis associated with venous TT in RCC has been well documented. Studies have reported a 40–50% recurrence rate among RCC patients with venous TT, underscoring the substantial oncological burden and the urgent need for enhanced risk stratification strategies [[19], [20], [21]]. A key innovation of this study lies in its focus on the radiomic analysis of the TT, distinguishing it from prior radiomic studies that predominantly focused on the primary tumor. The primary tumor RS + TT RS developed in this study may offer a noninvasive tool to enhance risk stratification for RCC patients with venous TT. It enables clinicians to identify high-risk patients who may benefit from intensified follow-up regimens or adjuvant therapies. Furthermore, radiomic-based risk scores can complement existing clinicopathological models, refine preoperative surgical planning, and facilitate shared decision-making with patients. Beyond this, integration of radiomics with emerging genomic or transcriptomic data could lay the foundation for comprehensive precision oncology strategies in RCC.

RCC is characterized by significant intratumoral heterogeneity driven by spatial and functional niches [22,23]. Recent multiregion whole-exome sequencing studies have shown that venous TTs often harbor viable tumor cells with distinct mutational profiles compared with the primary tumor. Notably, up to 22% of functional somatic variants differ between the primary tumor and thrombus. This genetic divergence may contribute to the distinct biological behavior and metastatic potential of the thrombus [24]. At the transcriptomic level, differential gene expression between the primary tumor and thrombus further supports their distinct roles in RCC progression. TTs exhibit upregulated pathways associated with proliferation, survival, and invasion, which may be less prominent in the primary tumor. For instance, the PI3K-AKT-mTOR signaling pathway is often more active within thrombi, potentially explaining their aggressive behavior and propensity for metastasis [25]. Additionally, the activation of gene signatures related to epithelial-mesenchymal transition and angiogenesis in thrombi underscores their involvement in vascular invasion and metastasis formation [26]. These molecular characteristics, which could be captured by radiomics, offer a noninvasive means to predict recurrence and inform clinical decision-making.

Our work has several notable strengths. First, unlike previous studies, we employed three-dimensional features, providing a more comprehensive representation of the tumor’s intrinsic characteristics. Second, the use of publicly available software facilitates external validation by other institutions. Third, we addressed scanner variability in multicenter studies using B-ComBat harmonization. Moreover, our design ensures interobserver consistency by involving physicians with varying levels of experience in assessing a representative subset of cases.

Several limitations of this study should be acknowledged. First, its retrospective design introduces inherent limitations, including potential biases and lack of control over data consistency. Additionally, the study had a relatively small sample size and lacked an external validation dataset. We tried to alleviate this issue by having a test cohort. Second, we did not evaluate molecular features based on genomics or proteomics. Further research integrating radiogenomics or multimodal approaches are needed to provide a comprehensive, system-level analysis of tumor characteristics. Third, this study was limited to contrast-enhanced CT imaging. Given that magnetic resonance imaging (MRI) provides superior soft-tissue contrast, an evaluation of TT using MRI-based radiomics could offer additional insights and should be considered in future studies. Finally, we intentionally excluded an overall survival analysis due to the considerable heterogeneity in patient management following recurrence, which could complicate the interpretation of long-term radiomic results.

## 5 Conclusions

Our study demonstrates the potential of TT radiomics in predicting recurrence in RCC. TT radiomic features extracted from preoperative CT scans significantly improved predictive performance over clinical models alone and provided greater net benefit than primary tumor radiomics. These findings highlight the importance of TT profiling as a noninvasive complement to traditional risk stratification. Validation in larger, multicenter cohorts is necessary to confirm these results and support clinical implementation.

  ***Author contributions:*** Vitaly Margulis had full access to all the data in the study and takes responsibility for the integrity of the data and the accuracy of the data analysis.

  *Study concept and design*: Khene, Bhanvadia, Tachibana, Gaston, Woldu, Bensalah, Lotan, Margulis.

*Acquisition of data*: Khene, Bhanvadia, Sharma, Graber, Trevino, Bingham, Bertail, Fleury.

*Analysis and interpretation of data*: Khene, Bhanvadia, Tachibana.

*Drafting of the manuscript*: Khene, Bhanvadia, Tachibana.

*Critical revision of the manuscript for important intellectual content*: None.

*Statistical analysis*: Khene, Bhanvadia, Tachibana.

*Obtaining funding*: None.

*Administrative, technical, or material support*: None.

*Supervision*: Bensalah, Lotan, Margulis.

*Other*: None.

  ***Financial disclosures:*** Vitaly Margulis certifies that all conflicts of interest, including specific financial interests and relationships and affiliations relevant to the subject matter or materials discussed in the manuscript (eg, employment/affiliation, grants or funding, consultancies, honoraria, stock ownership or options, expert testimony, royalties, or patents filed, received, or pending), are the following: Yair Lotan is a consultant for Nanorobotics, C2I Genomics, Photocure, AstraZeneca, Merck, Fergene, Abbvie, Nucleix, Ambu, Seattle Genetics, Hitachi, Ferring Research, Verity Pharmaceutics, Virtuoso Surgical, Stimit, Urogen, Vessi Medical, CAPs Medical, Xcures, BMS, Nonagen, Aura Biosciences, Inc., Convergent Genomics, Pacific Edge, Pfizer, Phinomics Inc, CG Oncology, Uroviu, On target Lab, Promis Diagnostics, Valar labs, and Uroessentials. Zine-Eddine Khene received financial support through grants from the Servier Institute for Interdisciplinary Studies.

  ***Funding/Support and role of the sponsor:*** None.
