# Supplementary material for: Radiomic Profiling of Tumor Thrombus for Predicting Recurrence in Renal Cell Carcinoma
Source: Eur Urol Open Sci. 2025 Jul 25;79:1–8. doi: 10.1016/j.euros.2025.06.005 (PMC12314389; doi:10.1016/j.euros.2025.06.005)

**Supplementary material**

**Supplementary Figure 1. Representative Volumes of Interest (VOIs) for Primary Tumor and Tumor Thrombus.** (A) Preoperative CT scan of a 52-year-old man with clear cell renal cell carcinoma and a tumor thrombus extending into the inferior vena cava. (B) Volume of interest (VOI) delineating the primary tumor. (C) VOI delineating the tumor thrombus. (D) Combined VOI including both the primary tumor and the tumor thrombus.

**
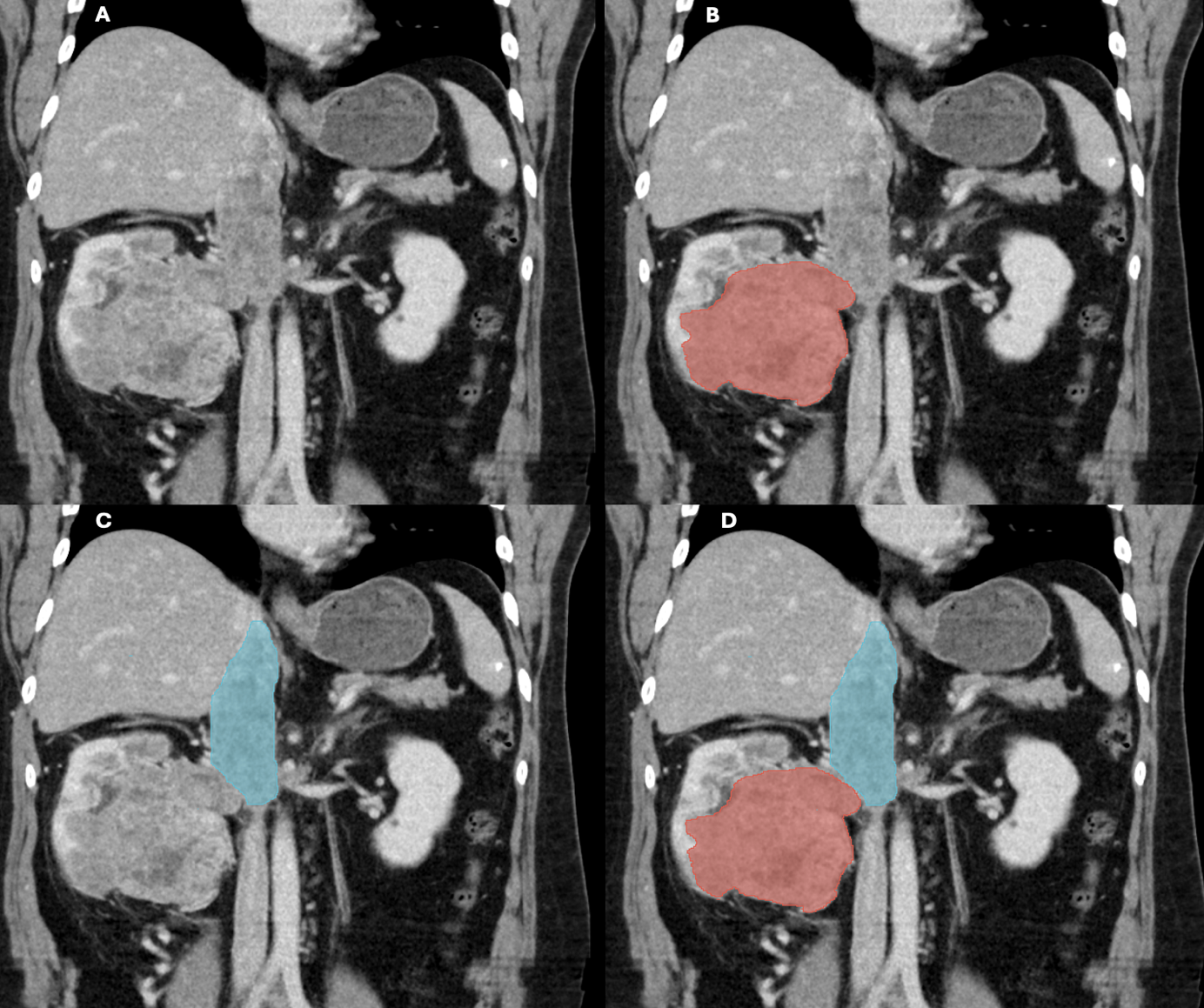
**

**Supplementary Figure 2. Kaplan-Meier curves for disease-free survival across the dataset: training and test set. (p = 0.94)**

**
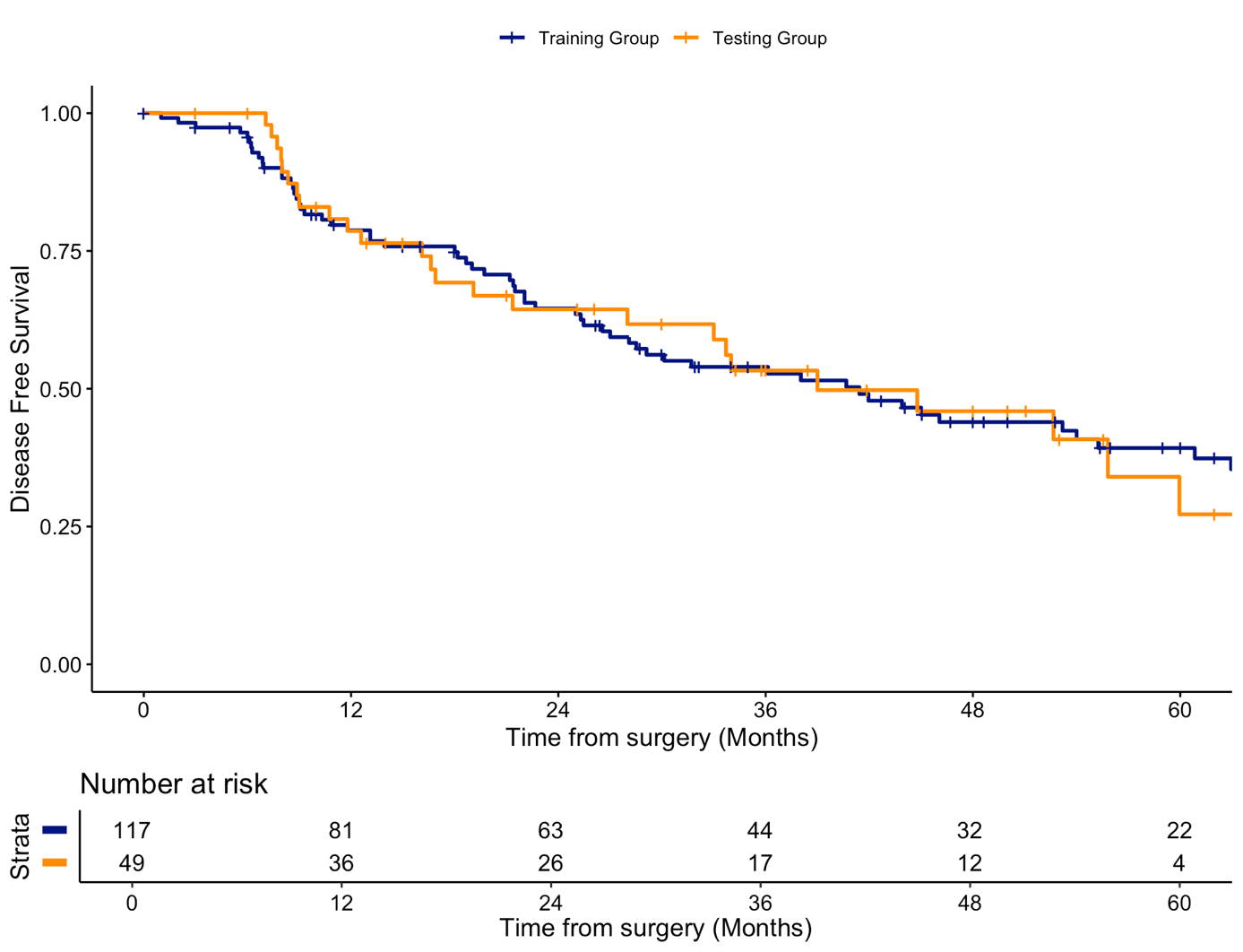
**

**Supplementary Table 1. CT Scanners and Parameters**

| **N = 166** | |
| --- | --- |
| **CT Scanner Vendor** |  |
| **GE MEDICAL SYSTEMS** | 64 |
| **PHILIPS** | 5 |
| **SIEMENS** | 86 |
| **TOSHIBA** | 11 |
| **Tube Current (mA)** | 200 (135, 320) |
| **Tube Voltage (kV)** |  |
| **90** | 1 |
| **100** | 48 |
| **110** | 2 |
| **120** | 107 |
| **140** | 8 |
| **Slice Thickness (mm)** | 2.00 (1.25, 3) |
| **Median_Pixel_Spacing** | 0.80 (0.70, 0.87) |
| **Number of Acquired Slices** | 160.00 (93.00, 304.00) |

**Supplementary Table 2. Radiomics Features Extracted**

| Feature Category | Description | Numbers | Names |
| --- | --- | --- | --- |
| Shape Features | Describe the three-dimensional size and shape of the tumor | 14 | original_shape_Elongation, original_shape_Flatness, original_shape_LeastAxisLength, original_shape_MajorAxisLength, original_shape_Maximum2DDiameterColumn, original_shape_Maximum2DDiameterRow, original_shape_Maximum2DDiameterSlice, original_shape_Maximum3DDiameter, original_shape_MeshVolume, original_shape_MinorAxisLength, original_shape_Sphericity, original_shape_SurfaceArea, original_shape_SurfaceVolumeRatio, original_shape_VoxelVolume |
| First-Order Features | describe the distribution of voxel intensities within the tumor | 18 | original_firstorder_10Percentile, original_firstorder_90Percentile, original_firstorder_Energy, original_firstorder_Entropy, original_firstorder_InterquartileRange, original_firstorder_Kurtosis, original_firstorder_Maximum, original_firstorder_MeanAbsoluteDeviation, original_firstorder_Mean, original_firstorder_Median, original_firstorder_Minimum, original_firstorder_Range, original_firstorder_RobustMeanAbsoluteDeviation, original_firstorder_RootMeanSquared, original_firstorder_Skewness, original_firstorder_TotalEnergy, original_firstorder_Uniformity, original_firstorder_Variance |
| GLCM Features | describe the second-order joint probability function of the tumor | 24 | original_glcm_Autocorrelation, original_glcm_ClusterProminence, original_glcm_ClusterShade, original_glcm_ClusterTendency, original_glcm_Contrast, original_glcm_Correlation, original_glcm_DifferenceAverage, original_glcm_DifferenceEntropy, original_glcm_DifferenceVariance, original_glcm_Id, original_glcm_Idm, original_glcm_Idmn, original_glcm_Idn, original_glcm_Imc1, original_glcm_Imc2, original_glcm_InverseVariance, original_glcm_JointAverage, original_glcm_JointEnergy, original_glcm_JointEntropy, original_glcm_MCC, original_glcm_MaximumProbability, original_glcm_SumAverage, original_glcm_SumEntropy, original_glcm_SumSquares |
| GLSZM Features | quantify gray-level zones in the tumor | 16 | original_glszm_GrayLevelNonUniformity, original_glszm_GrayLevelNonUniformityNormalized, original_glszm_GrayLevelVariance, original_glszm_HighGrayLevelZoneEmphasis, original_glszm_LargeAreaEmphasis, original_glszm_LargeAreaHighGrayLevelEmphasis, original_glszm_LargeAreaLowGrayLevelEmphasis, original_glszm_LowGrayLevelZoneEmphasis, original_glszm_SizeZoneNonUniformity, original_glszm_SizeZoneNonUniformityNormalized, original_glszm_SmallAreaEmphasis, original_glszm_SmallAreaHighGrayLevelEmphasis, original_glszm_SmallAreaLowGrayLevelEmphasis, original_glszm_ZoneEntropy, original_glszm_ZonePercentage, original_glszm_ZoneVariance |
| GLRLM Features | quantifies gray level runs, which are defined as the length in number of pixels, of consecutive pixels that have the same gray level value. | 16 | original_glrlm_GrayLevelNonUniformity, original_glrlm_GrayLevelNonUniformityNormalized, original_glrlm_GrayLevelVariance, original_glrlm_HighGrayLevelRunEmphasis, original_glrlm_LongRunEmphasis, original_glrlm_LongRunHighGrayLevelEmphasis, original_glrlm_LongRunLowGrayLevelEmphasis, original_glrlm_LowGrayLevelRunEmphasis, original_glrlm_RunEntropy, original_glrlm_RunLengthNonUniformity, original_glrlm_RunLengthNonUniformityNormalized, original_glrlm_RunPercentage, original_glrlm_RunVariance, original_glrlm_ShortRunEmphasis, original_glrlm_ShortRunHighGrayLevelEmphasis, original_glrlm_ShortRunLowGrayLevelEmphasis |
| GLDM Features | quantifies gray level dependencies in the tumor | 14 | original_gldm_DependenceEntropy, original_gldm_DependenceNonUniformity, original_gldm_DependenceNonUniformityNormalized, original_gldm_DependenceVariance, original_gldm_GrayLevelNonUniformity, original_gldm_GrayLevelVariance, original_gldm_HighGrayLevelEmphasis, original_gldm_LargeDependenceEmphasis, original_gldm_LargeDependenceHighGrayLevelEmphasis, original_gldm_LargeDependenceLowGrayLevelEmphasis, original_gldm_LowGrayLevelEmphasis, original_gldm_SmallDependenceEmphasis, original_gldm_SmallDependenceHighGrayLevelEmphasis, original_gldm_SmallDependenceLowGrayLevelEmphasis |
| NGTDM Features | quantifies the difference between a gray value and the average gray value of its neighbours within a certain distance | 5 | original_ngtdm_Busyness, original_ngtdm_Coarseness, original_ngtdm_Complexity, original_ngtdm_Contrast, original_ngtdm_Strength |
| Laplacian of Gaussian Features | Extracted at multiple scales (σ = 1 to 5): Each scale will have its own set of first-order, GLCM, GLSZM, GLRLM, GLDM, and NGTDM features | | |
| Wavelet Features | For each wavelet decomposition (LLL, LLH, LHL, LHH, HLL, HLH, HHL, HHH): Shape, first-order, GLCM, GLSZM, GLRLM, GLDM, NGTDM features | | |

***Radiomics Procedure***

Volumes of interest (VOIs) of the tumour were semiautomatically segmented using the GrowCut segmentation method implemented in the publicly available 3D Slicer software. GrowCut is an interactive region-growing segmentation method. Using the GrowCut method, the VOI is initially delineated. Then, we can meticulously edit the boundary of the regions of interest (ROIs) slice-by-slice by erasing or drawing the mask manually to improve the alignment of the ROIs with the tumour outlines. As a result, a VOIs was defined for each patient for radiomics feature extraction.

In this study, 1316 radiomics features were extracted from each defined VOIs using the *PyRadiomics* platform. As part of image preprocessing, images were resampled to isotropic voxels with 1-mm sides using a B Spline interpolator. Target region intensity values were discretized using a bin width of 25. Aside from the original image, features were also extracted from wavelet and Laplacian of Gaussian (LoG)-filtered images. For the wavelet filter, each image was filtered using either a high- bandpass filter or a low-bandpass filter in the x, y and z directions, yielding 8 different combinations of decompositions. For the LoG filter, images were filtered using a 3D LoG filter implemented in SimpleITK and by changing sigma values to 5.0, 4.0, 3.0, 2.0 and 1.0 mm, yielding another 5 derived images. Detailed information about the feature names and mathematical formulas can be obtained from the *pyradiomics* documentation available at <http://pyradiomics.readthedocs.io/en/latest>.

**Supplementary Table 3: Radiomic Features Selected by LASSO Penalized Cox Regression Analysis for Predicting Disease-Free Survival**

| **A. Primary Tumor Model** |  |
| --- | --- |
| **Feature** | **Coefficient** |
| original_glrlm_LongRunEmphasis | 0.08792629 |
| wavelet-HLL_glszm_LowGrayLevelZoneEmphasis | -0.1176304 |
| wavelet-HHH_firstorder_Median | -0.01412892 |
| log-sigma-3-0-mm-3D_glcm_ClusterShade | -0.06218747 |
| log-sigma-5-0-mm-3D_firstorder_Mean | 0.09827446 |
| log-sigma-5-0-mm-3D_glcm_JointEnergy | -0.03455213 |
| log-sigma-5-0-mm-3D_glszm_LargeAreaLowGrayLevelEmphasis | 0.08436249 |
| **B. Tumor Thrombus Model** |  |
| **Feature** | **Coefficient** |
| original_firstorder_Entropy | 0.90120696 |
| wavelet-HLL_glcm_ClusterProminence | 0.180225871 |
| wavelet-HHH_firstorder_Skewness | -0.109647313 |
| log-sigma-1-0-mm-3D_glcm_ClusterTendency | 0.009883908 |
| log-sigma-5-0-mm-3D_firstorder_90Percentile | 0.069989499 |
| **C. Combined Model (Primary Tumor and Tumor Thrombus)** |  |
| **Feature** | **Coefficient** |
| wavelet-HHL_glrlm_LongRunEmphasis | 0.06082288 |
| wavelet-HHL_glrlm_RunVariance | 0.02756743 |
| wavelet-LLL_gldm_DependenceVariance | -0.02617441 |
| v_original_firstorder_Entropy | 0.89082967 |
| v_wavelet-HLH_firstorder_Kurtosis | 0.10767212 |
| v_wavelet-HHH_firstorder_Skewness | -0.01126227 |
| v_log-sigma-5-0-mm-3D_firstorder_90Percentile | 0.07016451 |

**Supplementary Table 4. Simplified Definitions for Selected Radiomic Features**

| **Feature** | **Simplified Explanation** |
| --- | --- |
| **Long Run Emphasis** | Measures the size of large, uniform areas in the tumor. Larger areas suggest more homogenous regions, potentially related to tissue density. |
| **Low Gray Level Zone Emphasis** | Focuses on dark or low-intensity areas within the tissue, which might indicate less active or less dense tumor areas. |
| **Median** | The middle brightness value, giving a sense of how "bright" or "dark" the overall tumor area is. |
| **Cluster Shade** | Indicates how uneven or 'lumpy' the texture is; higher values suggest irregular, complex textures within the tumor. |
| **Mean** | Shows the average brightness, providing an idea of general tissue density in the tumor. |
| **Joint Energy** | Measures how uniform the intensity is; high values mean the tissue is more even in appearance. |
| **Large Area Low Gray Level Emphasis** | Highlights large, darker areas within the tumor, which could correspond to necrotic or less active tissue regions. |
| **Entropy** | Captures complexity; high entropy means the area is very mixed, with a lot of variation in intensity, indicating a more complex structure. |
| **Cluster Prominence** | Highlights distinct patterns in tissue structure; higher values indicate prominent clusters, which might suggest organized tissue patterns |
| **Skewness** | Reflects the asymmetry of intensity values, indicating a shift towards either brighter or darker regions within the tissue. |
| **Cluster Tendency** | Shows how much nearby pixels group together, suggesting a level of tissue organization. |
| **90th Percentile** | The intensity level below which 90% of pixels fall, highlighting brighter parts within the tissue. |
| **Run Variance** | Indicates variability in the lengths of similar-intensity regions, capturing complexity across the area. |
| **Dependence Variance** | Measures how much pixel intensities depend on neighboring values, suggesting how structured or chaotic the area is. |
| **Kurtosis** | Shows how "peaked" or "sharp" the intensity distribution is, often related to how uniform or focused tissue characteristics are. |

**Supplementary Figure 3. Calibration Plots for Predicted Recurrence Probabilities at 36 Months in the Test Cohort**


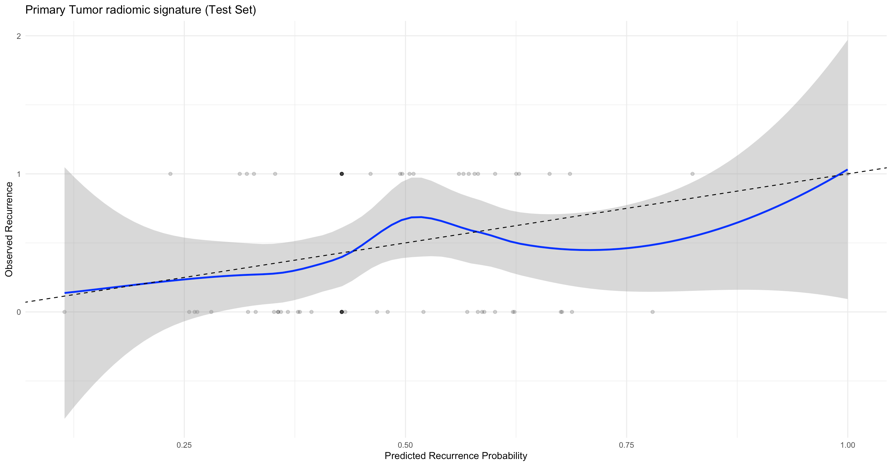


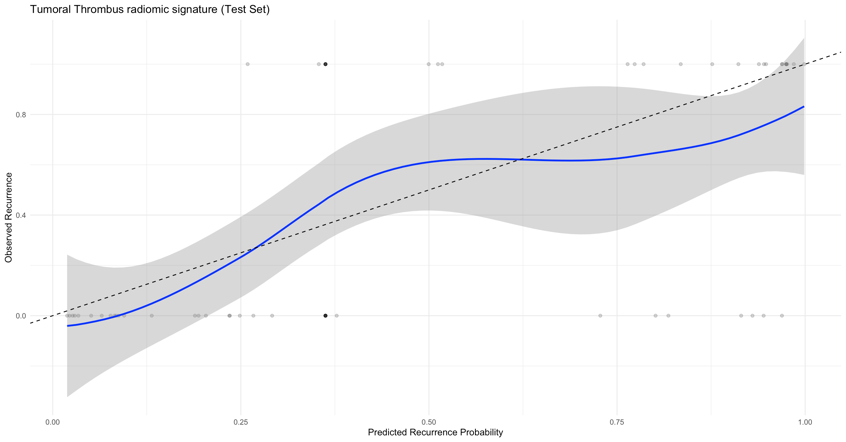


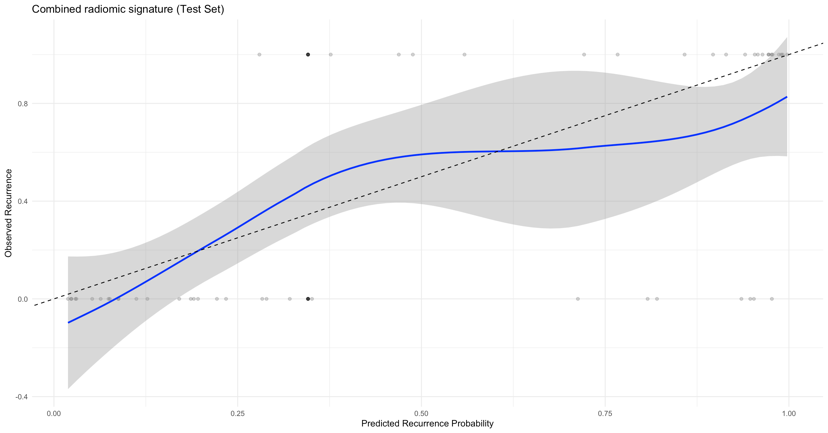


**Supplementary Figure 4 Decision Curve Analysis Demonstrating the Added Prognostic Value of Tumor Thrombus Radiomics in Combination with the Leibovich Score for RCC Recurrence Prediction**


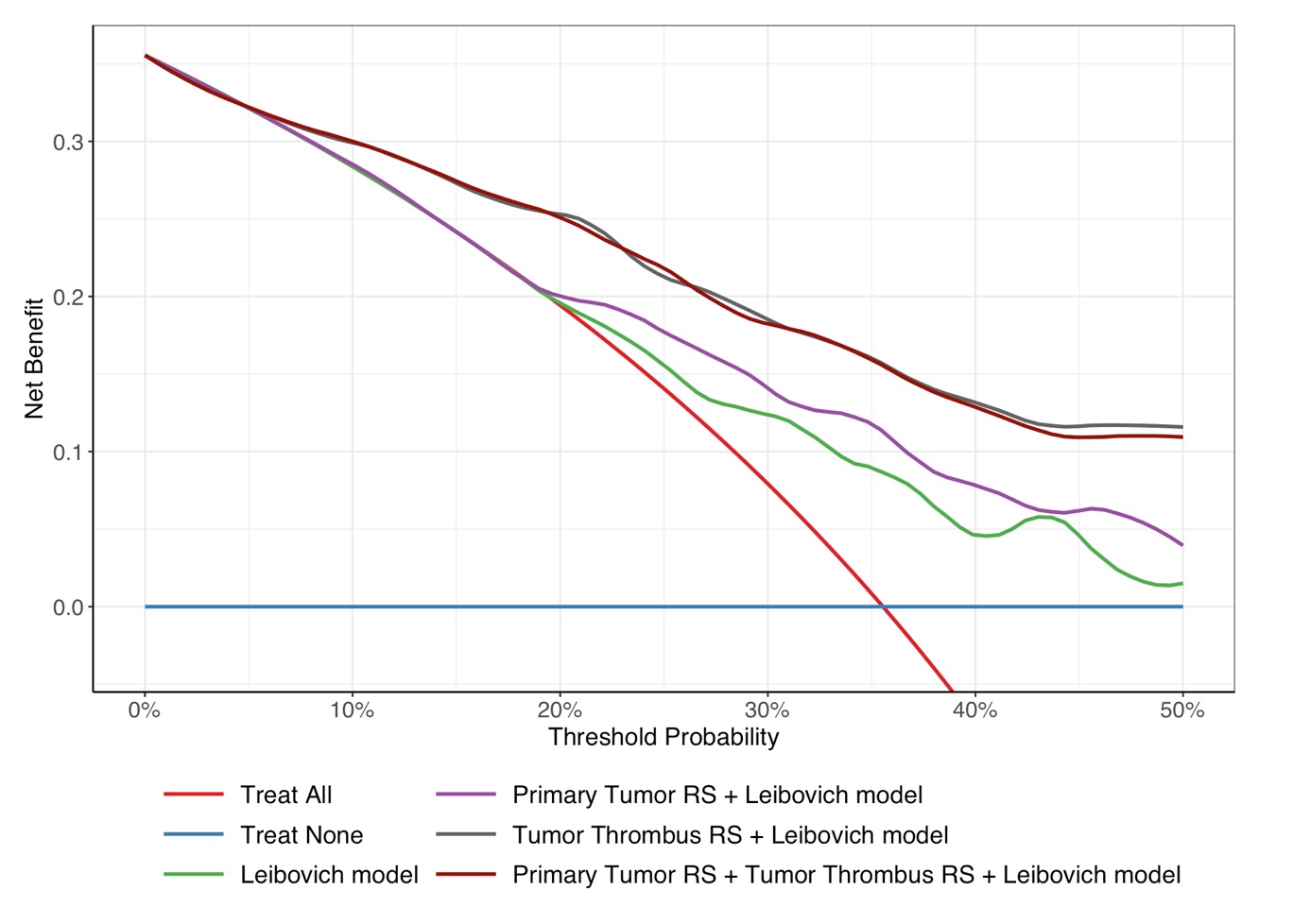

Supplement: Supplementary Data 1 [file mmc1.docx]
